# Investigating the KNDy Hypothesis in Humans by Coadministration of Kisspeptin, Neurokinin B, and Naltrexone in Men

**DOI:** 10.1210/jc.2016-1911

**Published:** 2016-07-05

**Authors:** Shakunthala Narayanaswamy, Julia K. Prague, Channa N. Jayasena, Deborah A. Papadopoulou, Maria Mizamtsidi, Amar J. Shah, Paul Bassett, Alexander N. Comninos, Ali Abbara, Stephen R. Bloom, Johannes D. Veldhuis, Waljit S. Dhillo

**Affiliations:** Section of Investigative Medicine (S.N., J.K.P., C.N.J., D.A.P., M.M., A.J.S., A.N.C., A.A., S.R.B., W.S.D.), Imperial College London, Hammersmith Hospital, London W12 ONN, United Kingdom; Statsconsultancy Ltd (P.B.), Amersham, Buckinghamshire HP7 9EN, United Kingdom; and Endocrine Research Unit (J.D.V.), Center for Translational Science Activities, Mayo Clinic, Rochester, Minnesota 55905

## Abstract

**Context:**

A subpopulation of hypothalamic neurons colocalize three neuropeptides, namely kisspeptin, neurokinin B (NKB), and dynorphin, collectively termed KNDy neurons. Animal studies suggest they interact to affect pulsatile GnRH release (KNDy hypothesis); kisspeptin stimulates, NKB modulates, and dynorphin (an opioid) inhibits.

**Objective:**

To investigate the KNDy hypothesis in humans, we assessed for the first time the effects of the coadministration of kisspeptin-54, NKB, and an opioid receptor antagonist, naltrexone, on LH pulsatility (surrogate marker for GnRH pulsatility) and gonadotropin release.

**Design, Setting, and Participants:**

This was an ethically approved prospective, single-blinded, placebo-controlled study. Healthy male volunteers (n = 5/group) attended our research facility for eight study visits.

**Intervention and Main Outcome Measure:**

After 1 hour of baseline blood sampling, participants received a different intervention at each visit: oral 50 mg naltrexone, 8-hour iv infusions of vehicle, 2.56 nmol/kg · h NKB, 0.1 nmol/kg · h kissspeptin-54 (KP) alone and in combination. Frequent blood sampling to measure plasma gonadotropins and sex steroids was conducted and LH pulsatility was determined using blinded deconvolution analysis.

**Results:**

All kisspeptin and naltrexone containing groups potently increased LH and LH pulsatility (*P* < .001 vs vehicle). NKB alone did not affect gonadotropins. NKB+KP had significantly lower increases in gonadotropins compared with kisspeptin alone (*P* < .01). Naltrexone+KP was the only group to significantly increase LH pulse amplitude (*P* < .001 vs vehicle).

**Conclusions:**

Our results suggest significant interactions between the KNDy neuropeptides on LH pulsatility and gonadotropin release in humans. This has important implications for improving our understanding of GnRH pulse generation in humans.

## Abstract (precis)

Co-administration of kisspeptin-54, NKB and an opioid antagonist naltrexone result in significant interactions to modulate LH pulsatility and gonadotrophin release in humans.

The field of reproductive neuroendocrinology has been evolving rapidly over the last decade since the discovery of the hypothalamic neuropeptide kisspeptin and its role in puberty and reproduction (1, 2). Kisspeptin has been shown to stimulate GnRH release and pulsatility. Recent evidence suggests that kisspeptin may not work alone but in combination with two other neuropeptides, neurokinin B (NKB) and dynorphin, collectively termed KNDy neurons (3), to affect GnRH release and pulsatility. NKB is a member of the tachykinin family and in humans is encoded by the tachykinin-3 (*TAC3*) gene and acts at the neurokinin-3 receptor (NK3R) encoded by the *TACR3* gene. Dynorphin is an opioid that acts at the κ-opioid receptor (KOR).

These three neuropeptides are found throughout the brain; however, they colocalize in a particular neuronal subpopulation in the arcuate nucleus (ARC) in mice (4), rats (5, 6), sheep (7), goats (8), and monkeys (9). The ARC is an important hypothalamic area that affects GnRH secretion and may represent the site of the elusive GnRH pulse generator (10). Multiunit activity (MUA) volleys near KNDy neurons in the ARC closely correspond with LH pulses (8). This has led to the concept of the KNDy hypothesis, which suggests that KNDy neurons in the ARC may interact to control GnRH release and pulsatility (4).

KNDy neuropeptides have been studied individually and affect LH secretion. Mutations in the genes encoding for kisspeptin, NKB, and their receptors (*KISS1*, *TAC3*, *KISS1R*, *TACR3*) result in hypogonadotrophic hypogonadism (1, 2, 11), indicating that both kisspeptin and NKB are critical for normal GnRH secretion in humans and subsequent puberty and reproduction. Kisspeptin has been shown to act on kisspeptin receptors (KISS1R) expressed on GnRH neurons to stimulate GnRH release (12–13), which subsequently stimulates gonadotropin secretion (14). However, NKB's effects have been more variable, with some studies showing stimulatory or inhibitory effects on gonadotropins. It is now clear that sex steroid milieu, pubertal status, and gender are important factors that govern the effects of NKB on gonadotropin release (15). Although the NKB receptor, NK3R, is not largely expressed on GnRH neurons (16–18), it is expressed on KNDy neurons in the ARC, and therefore, NKB is likely to act here to govern GnRH release (4, 5, 17).

Endogenous opioids have an inhibitory role on LH secretion. In particular the opioid dynorphin and agonists of its receptor, KOR, have been shown to inhibit LH secretion (4, 8, 19) as well as inhibiting MUA in the medial basal hypothalamus (8). Conversely, iv naloxone (an opioid receptor antagonist) increased MUA activity in the ARC as well as LH (20), and specific KOR antagonists have been shown to cause a rise in LH (21, 22). Thus, dynorphin is thought to act at the level of the hypothalamus to inhibit GnRH and subsequent gonadotropin secretion.

In summary, anatomical and functional data in animals suggest that KNDy neuropeptides play an important role to control GnRH release and pulsatility. In humans anatomical studies also suggest that the KNDy neuropeptides are colocalized in the infundibular nucleus (equivalent of the ARC in humans) (23, 24). However, no previous studies have investigated the effects of combined administration of the KNDy neuropeptides on gonadotropin release in humans.

Therefore, the aim of this study was to investigate the KNDy hypothesis in humans by assessing for the first time the effects of coadministration of kisspeptin-54, NKB, and an opioid receptor antagonist, naltrexone, on LH pulsatility (surrogate marker for GnRH pulsatility in humans) and gonadotropin release.

## Materials and Methods

### Ethics

Ethical approval was granted by the UK National Research Ethics Committee-Central London (Research Ethics Committee number 14/LO/1098). The study was performed in accordance with the Declaration of Helsinki. Written informed consent was obtained from all participants.

### Subjects

Healthy male volunteers aged between 18 and 45 years old were recruited from local newspaper advertisements (full details given in the Supplemental Methods).

### Peptides

Kisspeptin-54 was administered at 0.1 nmol/kg · h because this dose has been shown to cause a rise in LH in healthy men (14). NKB was administered at 2.56 nmol/kg · h because this dose was biologically active and well tolerated in previous studies (25).

Naltrexone hydrochloride (a nonselective opioid receptor antagonist) was administered as an oral tablet with water. Oral naltrexone is rapidly absorbed with peak plasma levels achieved after 1 hour (similar peak levels seen with iv administration) (26) and a half-life of 12 hours; thus, stable levels were achieved for most of the study period (26, 27). Oral naltrexone 50 mg was chosen because it has been shown in other human studies to cause a rise in LH at this dose (28, 29). Full details of kisspeptin-54, NKB and naltrexone are detailed in the Supplemental Methods.

### Protocol

**Figure 1.**
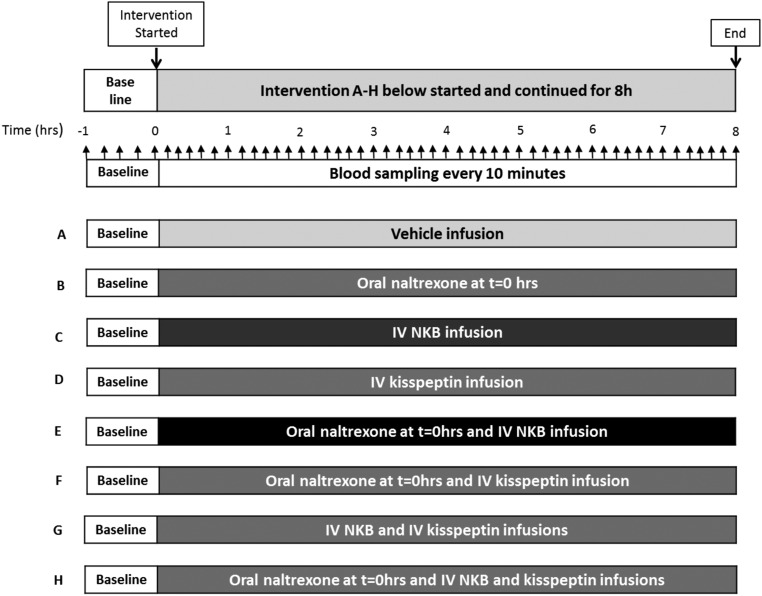
Study protocol diagram. Five healthy male participants attended for eight study visits at least a week apart and received a different treatment combination (A–H) in random order at each visit. After 1 hour of baseline blood sampling every 15 minutes, the treatments were initiated at t = 0 hour: naltrexone (single 50 mg tablet) swallowed with water or 8-hour infusions of vehicle, 2.56 nmol/kg · h NKB, or 0.1 nmol/kg · h kisspeptin-54 administered alone or in combinations (A–H below) in random order. Oral naltrexone is rapidly absorbed with peak plasma levels achieved after 1 hour and a half-life of 12 hours; thus, stable levels were achieved for most of the study period (26, 27). Ten-minute blood sampling for gonadotropins and T and hourly blood sampling for estradiol was commenced from the start of the treatment at t = 0 hour and continued for the duration of the 8-hour study period, after which the infusions were stopped and the study ended. Upward black arrows represent frequent blood sampling during the study.

Studies were conducted in our clinical investigation unit, and participants were asked to refrain from strenuous exercise, sexual activity, and alcohol consumption for the 24-hour period preceding each study visit. The subjects were blinded but not the investigators. Subjects arrived in the morning were cannulated in both forearms and asked to lie supine. The participants attended eight study visits at least a week apart and received a different treatment at each visit in random order. Figure 1 shows a summary of the protocol for each study visit and the treatments received (Figure 1, A–H). After 1 hour of baseline blood sampling, participants received either a naltrexone (NAL) 50-mg tablet to swallow with water or an iv infusions of vehicle (gelofusin), 2.56 nmol/kg · h NKB (NKB) or 0.1 nmol/kg · h kisspeptin-54 (KP) for 8 hours alone or in combination as outlined in the protocol diagram in Figure 1, A–H, and further explained in Supplemental Table 1, in random order. Regular blood sampling was commenced and continued for 8 hours to measure gonadotropins and sex steroids.

Because these peptides have never been coinfused in humans previously, we based our sample size calculation on a previous study in animals in which the effects of ip injections of NKB and kisspeptin alone and then in combination on gonadotropin release were investigated in male mice (Corander et al [30]).

### Analysis

Samples were analyzed for the measurement of LH, FSH, T, and estradiol using automated chemiluminescent immunoassays (Abbott Diagnostics). Reference ranges were as follows: LH, 4–14 IU/L; FSH, 1.5–8 IU/L; T, 10–28 nmol/L; and estradiol less than 190 pmol/L. The respective intraassay and interassay coefficients of variation for each assay were 4.1% and 2.7% (LH); 4.1% and 3.0% (FSH); 4.2% and 2.8% (total T); and 3.3% and 3.0% (estradiol). Analytical sensitivities were: 0.5 IU/L (LH), 0.05 IU/L (FSH), 2 nmol/L (total T), and 37 pmol/L (estradiol).

### Data analysis/statistical analysis

Statistical analysis of the data was performed by a statistician (P.B.). For the purposes of analysis, the eight treatment combinations were considered as eight different groups, rather than splitting into the three constituent peptides. Due to the relatively large number of combinations between pairs of groups, only comparisons with a value of *P* < .01 were considered statistically significant.

#### 

##### LH, FSH, T and estradiol statistical analysis.

The analyses were performed by modeling the response of LH, FSH, T, and estradiol over time for each treatment group. To allow for the repeat measurements over time, a multilevel linear regression analysis was used as well as two-level models with individual measurements nested within patients. Treatment group was considered to be a fixed effect. The differing relationships over time between treatment combinations were determined by examining the size of the interaction between treatment and time. A significant interaction would imply that the changes in outcome over time varied between treatment groups. To allow for a flexible curved relationship between time and the outcome variable, linear, squared, and cubic terms for time were included if found to improve the fit. The LH, FSH, T, and estradiol data suggest that the best fit came by including linear, squared, and cubic terms for time. LH and FSH results were found to have positively skewed distributions and thus were analyzed on the log scale. T and estradiol data were approximately normally distributed, and therefore, no data transformation was performed. In view of differing baseline sex steroid values between participants, T and estradiol results were analyzed as a change from baseline.

#### LH pulse number, amplitude, and approximate entropy (ApEn) analysis

J.D.V. used a previously described, blinded deconvolution method with 93% sensitivity and specificity to analyze LH pulsatility (31). J.D.V. calculated the LH pulse number, amplitude of each pulse, and the ApEn for each study visit. ApEn is a measure of orderliness of LH pulses (the lower the number, the more ordered the pulses in the time series, with zero denoting perfect orderliness) (31). To allow for repeat measurements, a statistical analysis was performed using multilevel linear regression. Two-level models were used with individual measurements nested within patients. Treatment group was considered to be a fixed effect.

## Results

The participant's characteristics, baseline gonadotropins, and sex steroid levels are shown in Supplemental Table 2: mean age ± SEM, 32.9 ± 3.9 years, and mean BMI, 23.8 ± 1.0. No participants reported any adverse effects.

### LH results

**Figure 2.**
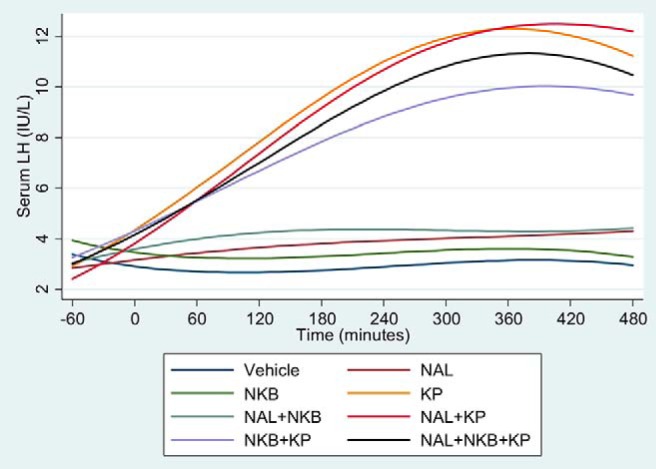
Effects of naltrexone (NAL), NKB, and kisspeptin (KP) on serum LH. Five healthy male participants attended for eight separate study visits at least a week apart and received vehicle, NAL, NKB, and KP alone, and in combination in random order. One-hour baseline blood sampling was conducted prior to starting the treatment. Ten-minute blood sampling was continued from the start of the treatment for 8 hours to measure gonadotrophins.

The results suggested a very distinct pattern dividing into two groups: those containing kisspeptin and those without. Treatment combinations containing kisspeptin (KP, NAL+KP, NKB+KP, NAL+NKB+KP) showed a large rise in LH, which was highly significant compared with vehicle (*P* < .001) over time up to around 360 minutes, after which the values levelled off (Figure 2).

In treatment combinations containing naltrexone but not kisspeptin, there was a smaller but highly significant rise in LH over time compared with vehicle in the NAL (*P* < .001) and NAL+NKB (*P* < .001) groups. The NKB group did not show a statistically significant rise in LH over time compared with vehicle (*P* = .75) (Figure 2). The individual LH results from all participants is shown in Supplemental Figure 1.

Given the sharp difference in LH, depending on whether kisspeptin was given, the focus of further analysis was on the difference between the kisspeptin-only group and the groups containing kisspeptin. There was a trend toward an increase in LH in the NAL+KP group compared with kisspeptin alone (*P* = .08). However, there was significantly lower increase in LH with the NKB+KP group (*P* = .006) compared with kisspeptin alone.

### LH pulsatility: number of LH pulses

**Figure 3.**
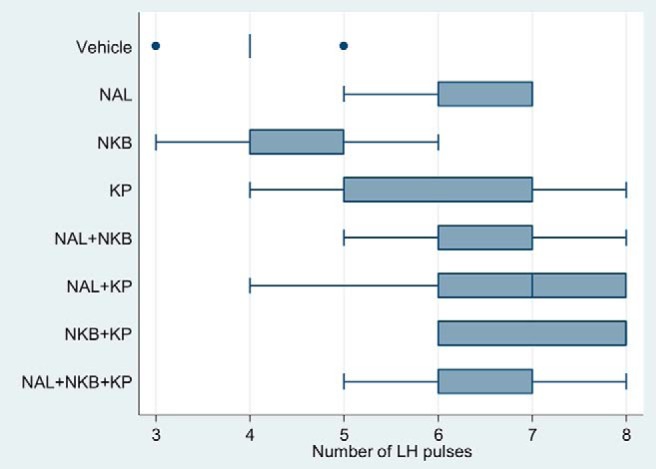
Effects of naltrexone (NAL), NKB, and kisspeptin (KP) on the number of LH pulses. Five healthy male participants attended for eight separate study visits at least a week apart and received vehicle, NAL, NKB, and KP alone and in combination in random order. One-hour baseline blood sampling was conducted prior to starting the treatment. Ten-minute blood sampling was continued from the start of the treatment for 8 hours to measure gonadotrophins. Deconvolution analysis was used to determine the number of LH pulses.

Most treatment groups achieved a significant increase in LH pulse number compared with vehicle (*P* < .001 for all treatment groups, except KP only [*P* = .007] and NKB [*P* = .55]) (Figure 3).

There was no significant difference in comparing the kisspeptin-containing groups with kisspeptin alone: NAL+KP (*P* = .23), NKB+KP (*P* = .04), and NAL+NKB+KP (*P* = .37).

### LH pulsatility: pulse amplitude

Specific comparisons between each group and vehicle showed that there was a significant rise in the LH pulse amplitude only in the NAL+KP group compared with vehicle (*P* = .001) (Supplemental Figure 2).

### LH pulsatility: ApEn

**Figure 4.**
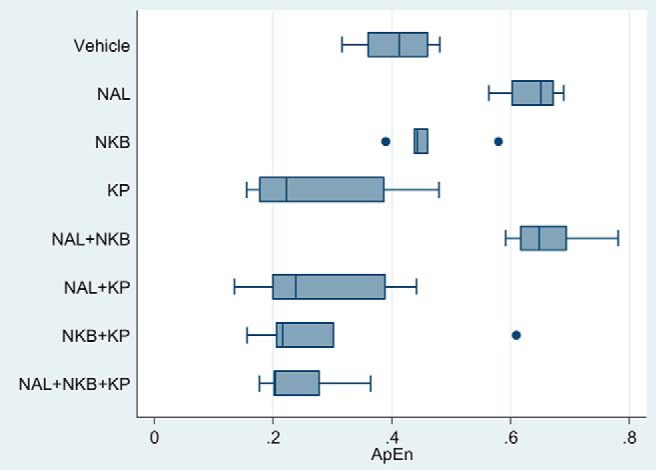
Effects of naltrexone (NAL), NKB, and kisspeptin (KP) on ApEn. Five healthy male participants attended for eight separate study visits at least a week apart and received vehicle, NAL, NKB, and KP alone and in combination in random order. One-hour baseline blood sampling was conducted prior to starting the treatment. Ten-minute blood sampling was continued from the start of the treatment for 8 hours to measure gonadotrophins. Deconvolution analysis was used to determine ApEn for each study. The ApEn is a measure of orderliness of LH pulses (the lower the number, the more ordered the pulses in the time series).

ApEn is a measure of orderliness of LH pulses (the lower the number, the more ordered the pulses) (31). The ApEn for each treatment group is shown in Figure 4. All kisspeptin-containing groups compared with vehicle showed a trend toward lower ApEn values, indicating more ordered pulses, but this reached the 1% significance level only for the NAL+NKB+KP group (NAL+NKB+KP, *P* < .001; KP, *P* = .02, NAL+KP, *P* = .02; NKB+KP, *P* = .04 vs vehicle).

In contrast, naltrexone-containing groups not containing kisspeptin had significantly higher ApEn compared with vehicle (NAL, *P* < .001; NAL+NKB, *P* < .001), indicating less ordered pulses. NKB alone had no significant effects on ApEn compared with vehicle (*P* = .28).

### Follicle-stimulating hormone

Similar to the LH results, any group containing kisspeptin (KP, NAL+KP, NKB+KP, NAL+NKB+KP) resulted in a highly significant rise in FSH compared with vehicle (*P* < .001) (Supplemental Figure 3).

In the nonkisspeptin-containing groups, only NAL+NKB showed a significant rise in FSH compared with vehicle (*P* = .002); the NAL alone group showed a trend toward a rise in FSH compared with vehicle, but this did not reach the 1% significance level (*P* = .03). NKB alone had no significant effects on FSH release compared with vehicle (*P* = .99). The individual FSH results from all participants are shown in Supplemental Figure 4.

Specific comparisons between the kisspeptin containing groups showed no significant difference with NAL+KP (*P* = .44) compared with kisspeptin alone. However, similarly to LH, there was significantly lower increase in FSH with the NKB+KP group compared with kisspeptin alone (*P* = .003).

### Testosterone

The rise in T levels in the treatment groups showed a similar pattern to the rise in LH in each group (Supplemental Figure 5). There was a significant rise in T compared with vehicle for the groups NAL, KP, and NAL+KP (*P* < .001 vs vehicle) and a trend toward a rise in T over vehicle but did not reach the 1% significance level in the NKB+KP (*P* = .04) and NAL+NKB+KP (*P* = .03) groups. There was no significant difference in T compared with vehicle in the remaining groups (NAL+NKB, *P* = .12; NKB, *P* = .2).

Specific comparisons between the kisspeptin-containing groups showed that the NKB+KP group resulted in a lower rise in T (similar to the lower rise in LH in this group) compared with KP alone (*P* = .01). In addition, the NAL+KP group showed a trend toward a greater rise T (similar to the trend toward a greater rise in LH in this group) compared with KP only (*P* = .02). The individual T results are shown in Supplemental Figure 6.

### Estradiol

The rise in estradiol levels in the treatment groups showed a similar pattern to the rise in T in each group (Supplemental Figure 7). The KP and NAL+NKB groups showed a significant increase in estradiol levels compared with vehicle (*P* = .001). Other groups showed a trend towards increasing estradiol compared to vehicle but did not reach the 1% significance level (NAL, *P* = .02; NAL+KP, *P* = .14; NKB+KP, *P* = .07; NAL+NKB+KP, *P* = .08). The NKB group did not show a rise in estradiol compared with vehicle (*P* = .50).

Specific comparisons between the kisspeptin-containing groups showed a trend toward a lower estradiol in the NKB+KP group compared with kisspeptin alone (*P* = .12) and a trend toward a higher estradiol level in the NAL+KP group compared with kisspeptin alone (*P* = .05).

## Discussion

Since the emergence of the KNDy neuron hypothesis for regulating GnRH secretion and pulsatility, animal studies have examined the interactions between the three neuropeptides. In this study we investigated the KNDy hypothesis in humans for the first time by coadministration of kisspeptin, NKB, and an opioid receptor antagonist, naltrexone, on gonadotropin release and LH pulsatility in healthy male volunteers.

Although both kisspeptin and naltrexone administered alone increased LH, their combined administration did not significantly increase LH secretion or LH pulsatility above that of kisspeptin alone. It is possible that kisspeptin alone had maximally stimulated LH release and hence no further increase occurred with coadministration of NAL+KP. However, only the NAL+KP group caused a significant rise in LH pulse amplitude, implicating opioid inhibition in limiting the kisspeptin induced rise in LH pulse amplitude. Animal studies have previously shown that pretreatment with a KOR antagonist unmasked the LH effects of senktide (NK3R agonist) (32, 33). In our study, there were no effects of coadministration of an opioid antagonist with NKB.

The data from volunteers who received combined NKB+KP revealed a novel interaction between NKB and KP in humans. Our data suggest that coadministration of NKB limited the ability of kisspeptin to stimulate a rise in LH and FSH.

It is interesting to speculate about the mechanism by which coadministration of NKB may have limited the ability of kisspeptin to stimulate a rise in LH in this human study. It is obviously not possible to determine changes in hypothalamic expression of neuropeptides after the acute hormone infusion in humans. However, studies in rats show that intra-ARC administration of senktide to ovariectomized and estradiol-treated rats resulted in a reduction in GnRH1 and KISS1R mRNA expression in the medial preoptic area (33). This suggests that ARC NKB may suppress GnRH secretion directly by reducing GnRH transcription but also indirectly by reducing KISS1R (33). Therefore, one could tentatively propose a mechanism in which NKB in the presence of an abundance of kisspeptin acts to inhibit GnRH secretion by affecting GnRH and KISS1R transcription to reduce the actions of kisspeptin on gonadotropin release.

NKB and kisspeptin have never been coinfused in humans previously, and relatively few animal studies have investigated the effects of peripheral coadministration of NKB+KP. One previous study in male mice showed that ip coadministration of NKB+KP resulted in an approximately 20% lower rise in LH compared with when ip KP was administered alone (30). Consistent with this in the same study, the authors investigated the interaction between NKB+KP in hypothalamic explants. They showed that NKB caused no increase in GnRH, whereas kisspeptin increased GnRH; however, coadministration of NKB+KP resulted in no increase in GnRH above basal state (30), indicating that NKB inhibited the kisspeptin induced rise in GnRH. This is similar to our findings in which coadministration of NKB+KP led to a lower rise of LH and FSH when compared with kisspeptin administration alone. The only other study that has investigated the effects of peripheral coadministration of NKB+KP was performed by Ramaswamy et al (34). They showed in GnRH primed juvenile rhesus monkeys that a continuous iv infusion of senktide or vehicle for 48 hours followed by iv kisspeptin-10 resulted in a similar rise in LH. However, this latter protocol was different from our study because a 48-hour infusion of high-dose senktide was administered to cause NK3R desensitization prior to kisspeptin treatment, unlike in our study in which NKB+KP were coadministered acutely over an 8-hour study period.

Another important consideration is whether sex steroid-negative feedback could have influenced our interpretation of the gonadotropin results (35) because the men in our study had normal testes that could respond to the increases in LH secretion observed in some groups. Our major findings were as follows: 1) alone and in combination, KP- and NAL-treated groups demonstrated an increase in LH and LH pulsatility vs vehicle groups and 2) NKB+KP demonstrated attenuated gonadotropins vs KP alone. If these findings were due to steroid-negative feedback, then we would have expected to observe a reduction in T and estradiol in the KP- and NAL-treated groups vs vehicle (therefore diminishing negative feedback) and to observe an increase in T and estradiol in the NKB+KP groups vs KP alone (therefore increasing negative feedback effects). However, neither of these hypotheses were evident in this time course. In fact, we observed that the increases in T and estradiol mirrored the gonadotropin changes in each group (Supplemental Figures 5–7). Together these data suggest that the results observed in this study were predominantly due to neuropeptide infusions and opioid receptor blockade on gonadotropin release rather than due to changes in steroid-negative feedback.

In this study we were also able to investigate the orderliness of the LH pulses (ApEn). Our results suggest that kisspeptin administration results in more ordered pulses, whereas opioid blockade results in more disordered pulses. In addition, kisspeptin may have a more dominant role in determining the orderliness of LH pulses as when kisspeptin is coadministered with naltrexone the effects are to produce more ordered pulses (and the ability of naltrexone to generate less ordered LH pulses are not observed).

The aim of our study was to investigate the KNDy hypothesis in humans for the first time, and our data reveal interesting and novel interactions after the coadministration of kisspeptin, NKB, and naltrexone on gonadotropin release and LH pulsatility in healthy male volunteers. However, we need to be cautious in extrapolating our findings to making definitive conclusions regarding the role of the KNDy neurons in humans. This is because the basis of the KNDy hypothesis is that the subpopulation of arcuate neurons that colocalize kisspeptin, NKB, and dynorphin interact together to affect pulsatile GnRH release. As such, many animal studies testing the KNDy hypothesis have used intracerebroventricular administration of peptides directly into the central nervous system. This is obviously not possible in humans, and we have used the peripheral administration of peptides, which may not have full access to the central nervous system, although the ARC is thought to have an incomplete blood-brain barrier and its KNDy neurons extend to the median eminence, which opens to the portal blood (36). In addition, kisspeptin and NKB have been demonstrated to have actions on kisspeptin and NK3R receptors on GnRH neurones extending into the median eminence and organum vasculosum lamina terminalis (9, 18, 37). Together this evidence suggests that peripherally administered kisspeptin and NKB can cross the blood-brain barrier sufficiently to affect GnRH neuronal function. Consistent with this, data in the current study show that kisspeptin and NKB were able to modulate LH pulsatility and gonadotropin release. Although kisspeptin, NKB, and naltrexone were administered peripherally, studies have shown that they have little direct effect on the pituitary gland but exert their effects on GnRH secretion above the level of the pituitary gland, which then subsequently determines pituitary gonadotropin secretion (9, 38, 39).

In this study we have been cautious in our interpretation of the results by using a 1% significance level (rather than the traditional 5% significance level) to define statistical significance. This was used on the advice our statistician (P.B.) to account for the relatively large number of combinations between pairs of groups. A limitation of this approach is that subtle interactions may have not been identified. However, the advantage of this approach is that the statistically significant findings presented in this study are robust.

In summary, our study has shown for the first time in humans the important interactions of KNDy signaling in regulating GnRH release and pulsatility (using LH as a surrogate marker). This has important implications not only for improving our understanding of GnRH pulse generation in humans but also in optimizing novel hormonal therapeutic agents to treat reproductive disorders.
